# Supplementary material for: Fatty Acids-Enriched Fractions of Hermetia illucens (Black Soldier Fly) Larvae Fat Can Combat MDR Pathogenic Fish Bacteria Aeromonas spp
Source: Int J Mol Sci. 2021 Aug 17;22(16):8829. doi: 10.3390/ijms22168829 (PMC8396364; doi:10.3390/ijms22168829)
Supplement: Supplementary file 1 [file ijms-22-08829-s001.zip › ijms-1285161-supplementary.pdf]

Time	a) *A. hydrophila*
12h	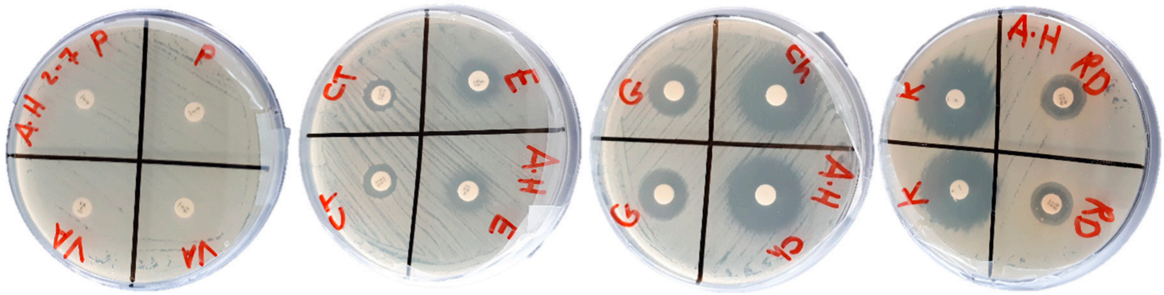
24h	b) *A. hydrophila*
	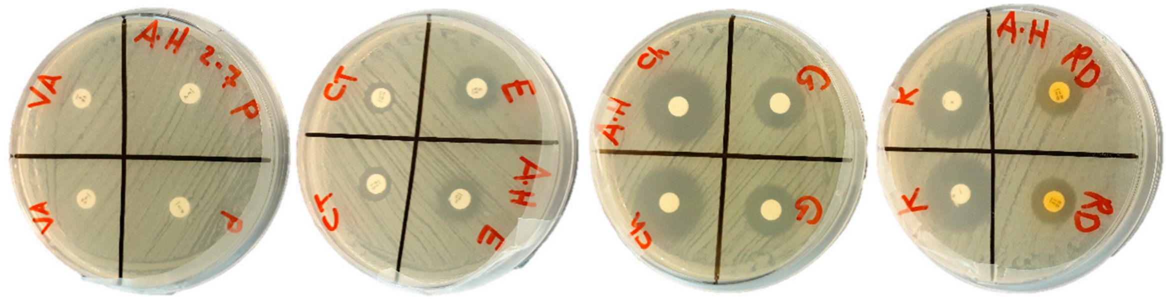
**12h**	c) *A. salmonicida*
	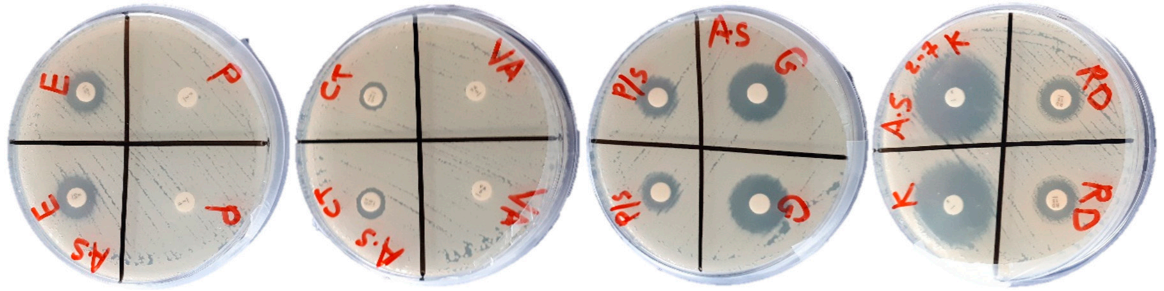
24h	d) *A. salmonicida*
	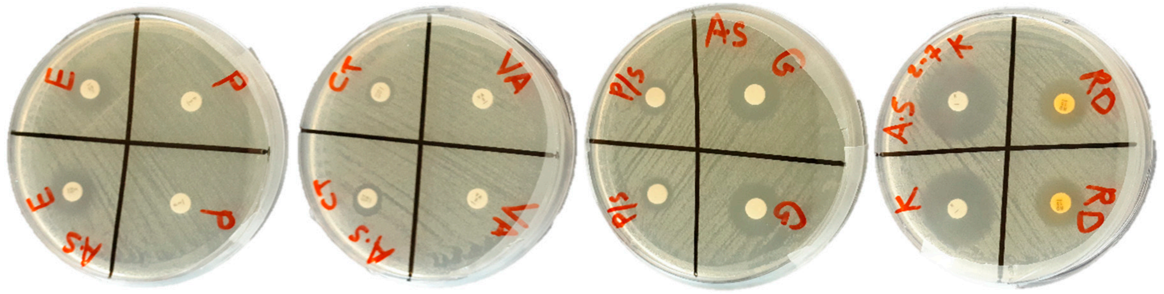

Supplementary Figure S1. Resistance patterns of *Aeromonas* spp to various classes of antimicrobial agents. Seven groups of antibiotics were tested against fish bacterial strains by the disc assay method. All antibacterial drugs were introduced to the Muller Hinton agar plates cultured with bacteria strains at density 10^8 (CFU/ml) and placed with gently pressing on the surface of the petri dishes agar. All discs were performed in duplicate and the results were the mean of IZD ±SD of three independent experiments.

Supplementary Table S1. Results of GC-MS analysis for AWME1 of BSFL fat..

S. No	RT	Area %	Name of the compound	Molecular formula	Mol. weight (g/mol)	Similarity %
1	5.918	0.62	1,2-Propanediol, 3-chloro-	$C_3H_7ClO_2$	110	82
2	10.405	0.31	Decanoic acid (Cpric acid)	$C_{10}H_{20}O_2$	172	89
3	11.866	3.18	Beta.-D-Glucopyranose, 1,6-anhydro-	$C_6H_{10}O_5$	162	92
4	12.33	19.32	Dodecanoic acid (Lauaric acid)	$C_{12}H_{24}O_2$	200	97
5	14.058	6.62	Tetradecanoic acid (Myristic acid)	$C_{14}H_{28}O_2$	228	97
6	14.683	0.42	Dodecanoic acid, ethenyl ester	$C_{14}H_{26}O_2$	226	79
7	14.756	0.3	Pentadecanoic acid	$C_{15}H_{30}O_2$	242	86
8	14.937	0.57	Octanoic acid, 2-dimethylaminoethyl ester	$C_{12}H_{25}NO_2$	215	89
9	15.188	0.3	Hexadecanoic acid methyl ester (Palmitic acid methyl ester)	$C_{17}H_{34}O_2$	270	89
10	15.286	3.03	cis-9-Hexadecenoic acid	$C_{16}H_{30}O_2$	254	96
11	15.412	26.19	n-Hexadecanoic acid (Palmitic acid)	$C_{16}H_{32}O_2$	256	96
12	15.585	3.08	Dodecanoyl chloride (Lauaric acid chloride)	$C_{12}H_{23}ClO$	218	75
13	15.917	0.19	Tridecanoic acid	$C_{14}H_{28}O_2$	228	83
14	16.02	1.36	Dodecanoic acid, 2,3-dihydroxypropyl ester (Glycerol alpha monolaurate)	$C_{15}H_{30}O_4$	274	82
15	16.139	1.35	9-Octadecenoic acid (Z)-, methyl ester(Methyl oleate)	$C_{19}H_{36}O_2$	296	89
16	16.258	0.37	Octadecanoic acid, methyl ester (Stearic acid methyl ester)	$C_{19}H_{38}O_2$	298	86
17	16.345	22.65	Octadec-9-eonic acid (Oleic acid)	$C_{18}H_34O_2$	282	95
18	16.435	5.93	Octadecanoic acid (Stearic acid)	$C_{18}H_{36}O_2$	284	93
19	16.724	0.37	9,12-Octadecadienoic acid (Z,Z)-(Linoleic acid)	$C_{18}H_{32}O_2$	280	91
20	17.003	0.82	Undecanal, 2-methyl-	$C_{12}H_{24}O$	184	82
21	17.115	0.16	Tetradecanoic acid, 2-hydroxy-1-(hydroxymethyl)ethyl ester(Myristic acid beta mono glyceride)	$C_{17}H_{34}O_4$	302	75
22	17.347	0.34	Eicosanoic acid (Arachidic acid)	$C_{20}H_{40}O_2$	312	88
23	17.663	0.32	Oleoyl chloride (Oleic acid chloride)	$C_{18}H_{33}ClO$	300	87
24	17.824	0.35	Ethanamine, 2,2'-oxybis[N,N-dimethyl-	$C_8H_{20}N_2O$	160	80
25	18.103	0.4	Hexadecanoic acid, 2-hydroxy-1-(hydroxymethyl)ethyl ester (Palmitic acid .beta.-monoglyceride)	$C_{19}H_{38}O_4$	330	92
26	18.551	0.32	Octadecanamide	$C_{18}H_{37}NO$	283	83
27	20.299	0.39	Dodecanoic acid, 1-(hydroxymethyl)-1,2-ethanediyl ester	$C_{27}H_{52}O_5$	456	72
28	21.134	0.34	Cholesta-3,5-diene	$C_{27}H_{44}$	368	75

Supplementary Table S2. Results of GC-MS analysis for AWME2 of BSFL fat.

S. No	RT	Area %	Name of the compound	Molecular formula	Mol. weight (g/mol)	Similarity %
1	6.04	5.26	1,2-Propanediol, 3-chloro-	$C_3H_7ClO_2$	110	97
2	6.27	1.18	2-Chloro-1,3-propandiol	$C_3H_7ClO_2$	110	77
3	7.42	3.47	1,2,3-propantriol (Glycerol)	$C_3H_8O_3$	92	95
4	10.405	0.26	n-Decanoic acid (Capric acid)	$C_{10}H_{20}O_2$	172	86
5	12.33	16.68	Dodecanoic acid (Lauric acid)	$C_{10}H_{20}O2$	200	97
6	14.058	5.56	Tetradecanoic acid (Myristic acid)	$C_{14}H_{28}O_2$	228	97
7	14.684	0.25	Dodecanoic acid, ethenyl ester	$C_{14}H_{26}O_2$	226	77
8	14.755	0.2	Pentadecanoic acid	$C_{15}H_{30}O_2$	242	94
9	14.941	1.24	Octanoic acid, 2-dimethylaminoethyl ester	$C_{12}H_{25}NO_2$	215	92
10	15.188	0.32	Hexadecanoic acid, methyl ester (Palmitic acid methyl ester)	$C_{17}H_{34}O_2$	270	88
11	15.286	3.05	cis-9-Hexadecenoic acid	$C_{16}H_{30}O_2$	254	96
12	15.414	22.01	n-Hexadecanoic acid (Palmitic acid)	$C_{16}H_{32}O_2$	256	96
13	15.858	0.18	Dodecanoic acid, 2-hydroxy-1-(hydroxymethyl) ethyl ester	$C_{15}H_{30}O_4$	274	75
14	15.918	0.18	Tridecanoic acid	$C_{13}H_{26}O_2$	214	87
15	16.137	1.36	Dodecanoic acid, 2,3-dihydroxypropyl ester (Glycerol .alpha.-monolaurate)	$C_{15}H_{30}O_4$	274	85
16	16.349	23.9	Octadec-9-enoic acid (Oleic acid)	$C_{18}H_{34}O_2$	282	95
17	16.438	5.74	Octadecanoic acid (Stearic acid)	$C_{18}H_{36}O_2$	284	94
18	16.538	0.22	13-Tetradece-11-yn-1-ol	$C_{14}H_{24}O$	208	77
19	16.724	0.21	9,12-Octadecadienoic acid (Z,Z)-(Linoleic acid)	$C_{18}H_{32}O_2$	280	89
20	17.002	1.21	N1-Isopropyl-2-methyl-1,2-propanediamine	$C_7H_{18}N_2$	130	83
21	17.114	0.51	Tetradecanoic acid, 2-hydroxy-1-(hydroxymethyl)ethyl ester (Myristic acid .beta.-monoglyceride)	$C_{17}H_{34}O_4$	302	78
22	17.344	0.46	Eicosanoic acid (Arachidic acid)	$C_{20}H_{40}O_2$	312	91
23	17.822	0.49	Octanoic acid, 2-dimethylaminoethyl ester	$C_{12}H_{25}NO_2$	215	87
24	18.101	0.94	Hexadecanoic acid, 2-hydroxy-1-(hydroxymethyl)ethyl ester (Palmitic acid .beta.-monoglyceride)	$C_{19}H_{38}O_4$	330	95
25	18.336	0.26	9-Octadecanoic acid (Z)-	$C_{18}H_{34}O_2$	282	88
26	18.55	0.48	Tetradecanamide	$C_{14}H_{29}NO$	227	92
27	18.616	0.12	Tritriacontane	$C_{33}H_{68}$	464	79
28	19.009	1.68	Oleoyl chloride	$C_{18}H_{33}ClO$	300	93
29	19.162	1.09	9-Octadecenoic acid, 1,2,3-propanetriyl ester, (E,E,E)-	$C_{57}H_{104}O_6$	884	91
30	19.906	0.27	Octadecanamide	$C_{18}H_{37}NO$	283	91
31	21.137	0.35	Cholesta-3,5-diene	$C_{27}H_{44}$	368	80

Supplementary Table S3. GC-MS analysis revealed that fatty acids were the major components of the AWME3 of BSFL fat.

S. No	RT	Area %	Name of the compound	Molecular formula	Mol. weight (g/mol)	Similarity %
1	3.798	0.11	2-Propanone, 1-hydroxy-	$C_3H_6O_2$	74	83
2	5.928	0.85	1,2-Propanediol, 3-chloro- (Glycerol .alpha.-chlorohydrin)	$C_3H_7ClO_2$	110	94
3	6.808	7.87	1,2,3-PROPANETRIOL (Glycerol)	$C_3H_8O_3$	92	96
4	10.403	0.3	n-Decanoic acid (Capric acid)	$C_{10}H_{20}O_2$	172	84
5	11.552	0.11	BENZENE, OCTYL-	$C_{14}H_{22}$	190	82
6	11.84	0.14	Beta.-D-Glucopyranose, 1,6-anhydro- (Levoglucosan)	$C_6H_{10}O_5$	162	77
7	12.331	17.66	Dodecanoic acid (Lauric acid)	$C_{12}H_{24}O_2$	200	97
8	13.923	0.13	2,4-Dodecadienal, (E,E)-	$C_{12}H_{20}O$	180	80
9	14.058	5.27	Tetradecanoic acid (Myristic acid)	$C_{14}H_{28}O_2$	228	97
10	14.683	0.27	Dodecanoic acid, ethenyl ester	$C_{14}H_{26}O_2$	226	77
11	14.753	0.2	Pentadecanoic acid	$C_{15}H_{30}O_2$	242	91
12	14.936	0.64	3-Cyclopentylpropionic acid, 2-dimethylaminoethyl ester	$C_{12}H_{23}NO_2$	213	92
13	15.187	0.23	Hexadecanoic acid, methyl ester (Palmitic acid methyl ester)	$C_{17}H_{34}O_2$	270	88
14	15.286	3.15	cis-9-Hexadecenoic acid	$C_{16}H_{30}O_2$	254	96
15	15.413	21.76	n-Hexadecanoic acid (Palmitic acid)	$C_{16}H_{32}O_2$	256	96
16	15.55	0.97	Dodecanoyl Chloride (Lauric acid chloride)	$C_{12}H_{23}ClO$	218	75
17	15.761	0.21	Cholest-5-en-3-ol (3.beta.)-, carbonochloridate (Cholesterol, chloroformate)	$C_{28}H_{45}ClO_2$	448	76
18	16.021	1.08	Dodecanoic acid, 2-hydroxy-1-(hydroxymethyl)ethyl ester (Lauric acid .beta.-monogly)	$C_{15}H_{30}O_4$	274	82
19	16.137	1.14	Oxiraneundecanoic acid, 3-pentyl-, methyl ester, cis-	$C_{19}H_{36}O_3$	312	85
20	16.35	26.28	Octadec-9-enoic acid (cis-oleic acid)	$C_{18}H_{34}O_2$	282	95
21	16.439	5.82	Octadecanoic acid (Stearic acid)	$C_{18}H_{36}O_2$	284	94
22	16.542	0.25	9,12-Hexadecadienoic acid, methyl ester	$C_{17}H_{30}O_2$	266	78
23	16.723	0.21	9,12-Octadecadienoic acid (Z,Z)-(Linoleic acid)	$C_{18}H_{32}O_2$	280	91
24	17.004	0.86	Undecanal, 2-methyl-	$C_{12}H_{24}O$	184	81
25	17.345	0.31	Eicosanoic acid (Arachidic acid)	$C_{20}H_{40}O_2$	312	90
26	17.82	0.23	Octanoic acid, 2-dimethylaminoethyl ester	$C_{12}H_{25}NO_2$	215	82
27	17.901	0.13	cis-9-Hexadecenal	$C_{16}H_{30}O$	238	83
28	18.101	0.49	Hexadecanoic acid, 2-hydroxy-1-(hydroxymethyl)ethyl ester	$C_{19}H_{38}O_4$	330	95
29	18.55	0.46	Octadecanamide	$C_{18}H_{37}NO$	283	92
30	19.01	1.46	Oleoyl chloride (Oleic acid chloride)	$C_{57}H_{104}O_6$	300	92
31	19.163	0.61	9-Octadecenoic acid, 1,2,3-propanetriyl ester, (E,E,E)-	$C_{57}H_{104}O_6$	884	89

32	20.3	0.44	Octadecanoic acid, 2,3-bis[(1-Oxotetradecyl)Oxy]propyl ester	$C_{49}H_{94}O_6$	778	77
33	21.136	0.36	Cholesta-3,5-Diene	$C_{27}H_{44}$	368	90

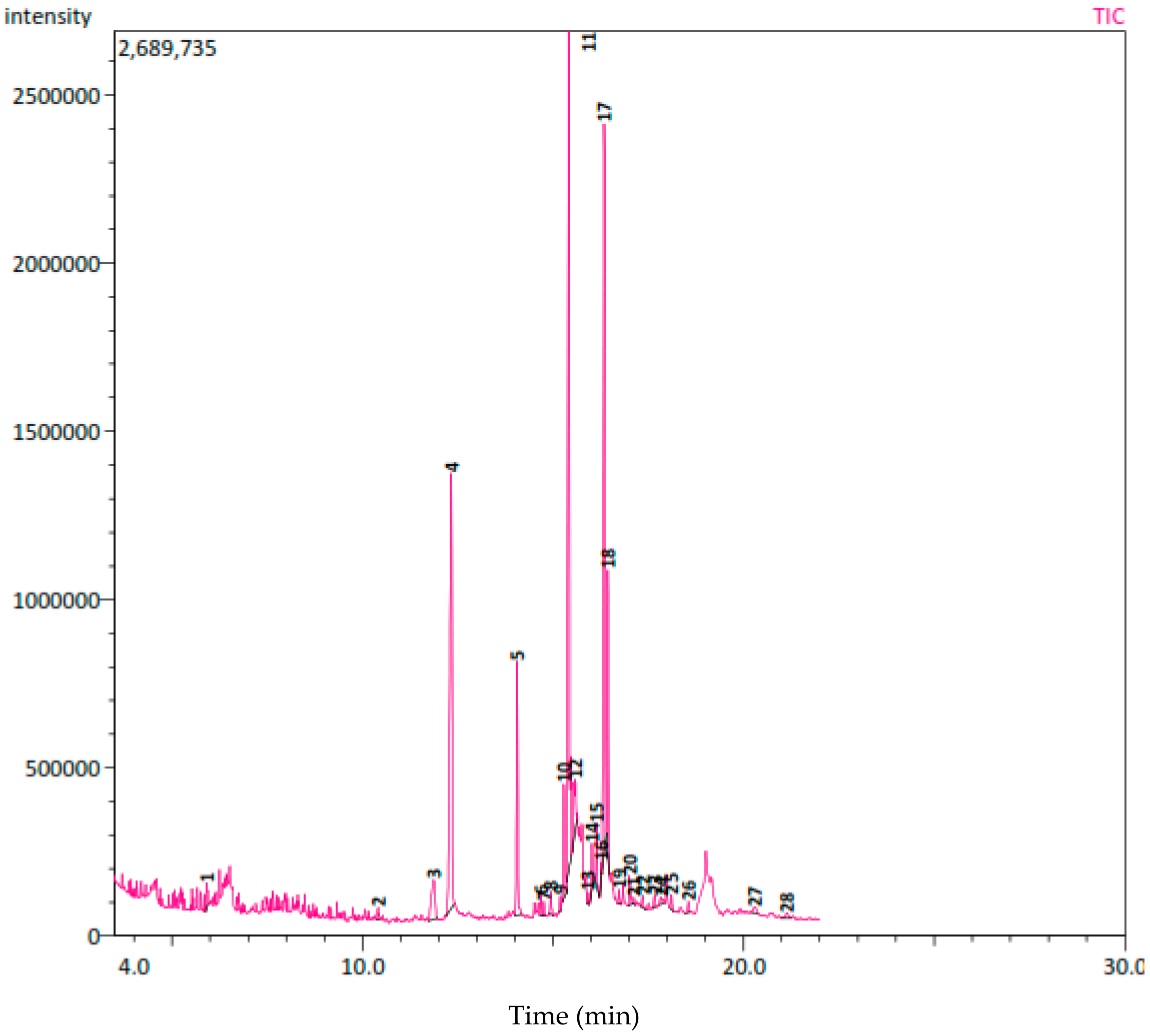

Supplementary Figure S2. GC-MS chromatogram of AWME1 from BSFL fat. The chemical composition formed of 28 compounds from the AWME1 of BSFL fat estimated by GC-MS and identification based on NIST-08 library.

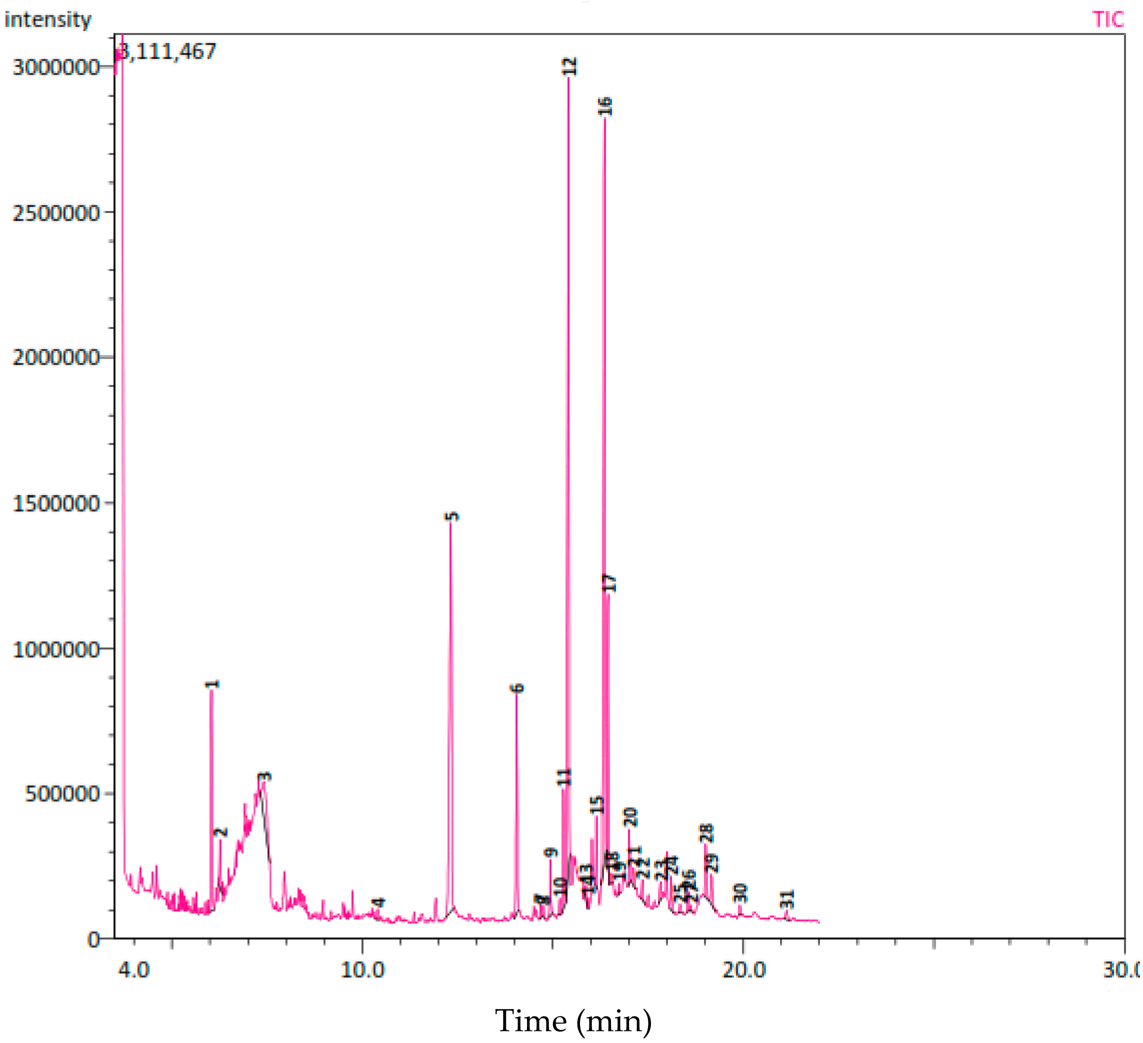

Supplementary Figure S3. GC-MS chromatogram of AWME2 from BSFL fat. The chemical composition formed of 31compounds from the AWME2 of BSFL fat estimated by GC-MS and identification based on NIST-08 library.

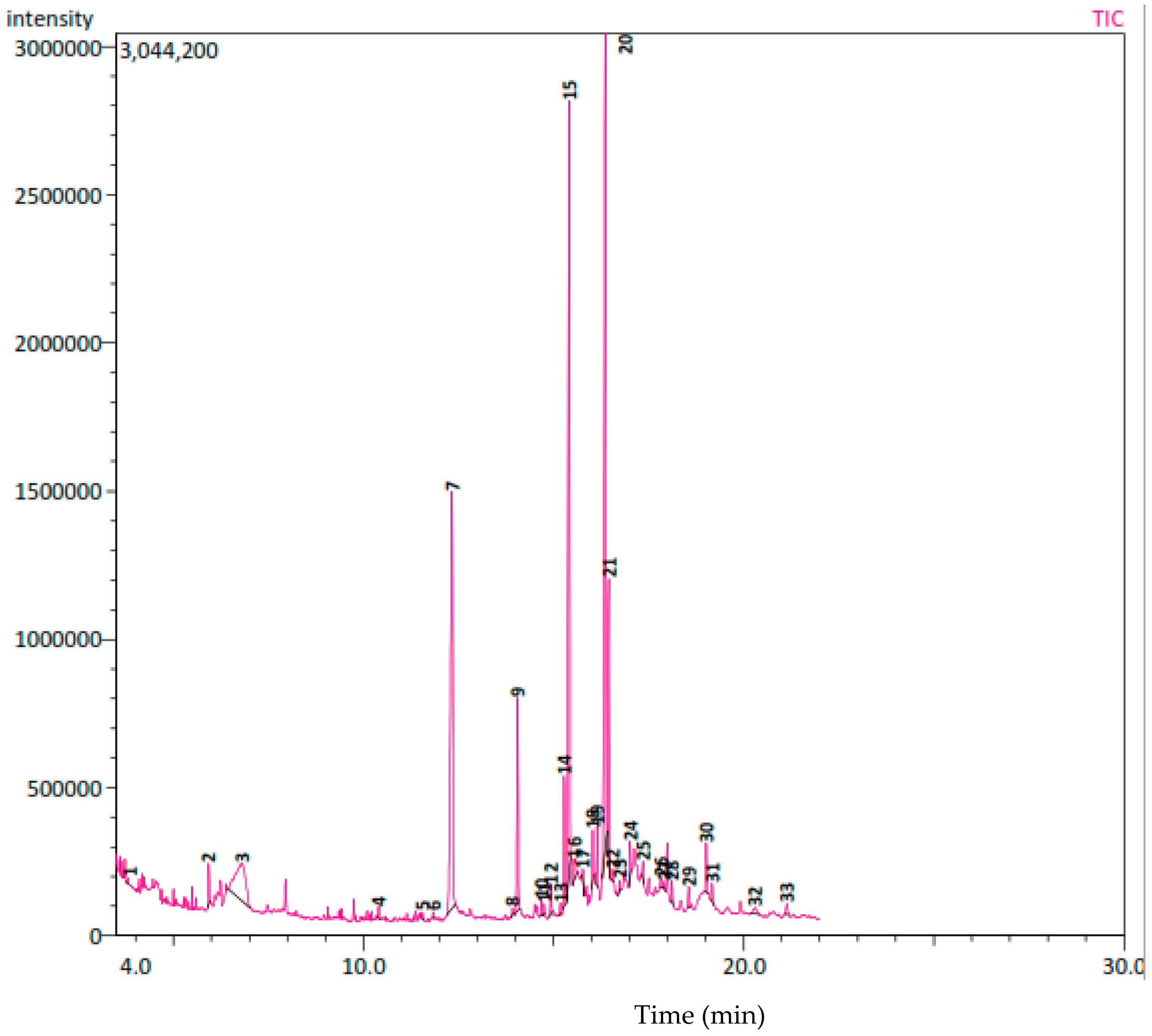

Supplementary Figure S4. GC-MS chromatogram of AWME3 from BSFL fat. The chemical composition formed of 33 compounds from the AWME3 of BSFL fat estimated by GC-MS and identification based on NIST-08 library.
